# Barriers to investing in cycling: Stakeholder views from England

**DOI:** 10.1016/j.tra.2017.11.003

**Published:** 2019-10

**Authors:** Rachel Aldred, Tom Watson, Robin Lovelace, James Woodcock

**Affiliations:** a Department of Planning and Transport, Faculty of Architecture and the Built Environment, Westminster University, 35 Marylebone Road, London NW1 5LS, United Kingdom; b Policy Studies Institute, Faculty of Architecture and the Built Environment, Westminster University, 35 Marylebone Road, London NW1 5LS, United Kingdom; c Institute for Transport Studies, University of Leeds, 34-40 University Road, Leeds LS2 9JT, United Kingdom; d UKCRC Centre for Diet and Activity Research (CEDAR), MRC Epidemiology Unit, University of Cambridge School of Clinical Medicine, Box 285 Institute of Metabolic Science, Cambridge Biomedical Campus, Cambridge CB2 0QQ, United Kingdom

## Abstract

**Background:**

Planners and politicians in many countries seek to increase the proportion of trips made by cycling. However, this is often challenging. In England, a national target to double cycling by 2025 is likely to be missed: between 2001 and 2011 the proportion of commutes made by cycling barely grew. One important contributory factor is continued low investment in cycling infrastructure, by comparison to European leaders.

**Methods:**

This paper examines barriers to cycling investment, considering that these need to be better understood to understand failures to increase cycling level. It is based on qualitative data from an online survey of over 400 stakeholders, alongside seven in-depth interviews.

**Results:**

Many respondents reported that change continues to be blocked by chronic barriers including a lack of funding and leadership. Participants provided insights into how challenges develop along the life of a scheme. In authorities with little consideration given to cycling provision, media and public opposition were not reported as a major issue. However, where planning and implementation have begun, this can change quickly; although examples were given of schemes successfully proceeding, despite this. The research points to a growing gap between authorities that have overcome key challenges, and those that have not.

## 1 Introduction

This paper analyses qualitative data from (a) a survey of 413 stakeholders sampled from across England and (b) supplementary in-depth interviews with seven cycling stakeholders. The analysis explores barriers to effective investment in cycling, and provides examples of how barriers can be overcome. Recommended measures to overcome these barriers at local and national levels are made, many of which may apply to other countries seeking to increase the level of cycling from a low base.

### 1.1 The need for investment in cycling

The benefits of a population shift to more cycling are well-established (Celis-Morales et al., 2017, Götschi et al., 2015, Sá et al., 2017). National and many local government organisations in England have for decades have stated aims to support and increase cycling (Aldred, 2012). So why are levels of cycling still so low? Increasingly research has highlighted fear of motor traffic as a major barrier to cycling (Jacobsen et al., 2009, Pooley et al., 2013). High quality cycling infrastructure can help to create transport systems in which people can cycle without the danger and stress of mixing with motor traffic (Transport for London, 2014, Pucher and Buehler, 2008). A recent systematic review (Aldred et al., 2016) found that people under-represented in UK cycling statistics, especially women and elderly people, tend to more strongly prefer cycling on infrastructure that is wholly or largely separated from motor traffic.

Evidence increasingly suggests that building such infrastructure along key desire lines can increase cycling levels (Goodman et al., 2014, Panter et al., 2016). Cycling interventions and policies can be highly cost-effective, providing not only transport benefits, but also health, business, and social benefits (Jensen et al., 2013). An analysis of transport interventions to boost walking and cycling, including data on health impacts, found 16 out of 17 were cost effective (Cavill et al., 2008). A review carried out for the UK’s Department for Transport (DfT) drew attention to the relatively low capital expenditure requirements for compact urban areas that enable walking and cycling (Rajé and Saffrey, 2016).

In England, how might cycling infrastructure be funded? There are several possibilities, with surface transport funding partially devolved to different governance levels. Local transport authorities are allocated funding blocks from DfT for local transport schemes and maintenance of existing systems. Excepting London where devolution is greater, DfT controls and/or directly funds schemes relating to major “strategic” roads, and some other larger and cross-boundary schemes. Funding for some medium-sized schemes is currently allocated via ‘Growth Deals’ from the Department for Business, Energy and Industrial Strategy (BEIS) to Local Enterprise Partnerships (LEPs), public-private planning organisations covering several local authority areas.^1^ Some cities have used European Union funding to implement pedestrian and cycling schemes.

Cycling infrastructure is cheap compared to main road upgrades and high-speed rail. However, it is expensive compared to the more traditional British approach of boosting cycling by encouragement, training and promotion (Golbuff and Aldred, 2011). A report for the UK’s All Party Parliamentary Cycling Group recommended a substantial increase, initially to a minimum of £10 per head and rising to £20 per head (Goodwin, 2015). This would bring UK spending on cycling towards the Dutch levels of £24 per person per year (British Cycling, 2016), a country that already has substantial infrastructural provision (Wardlaw, 2014). Currently only London, where many transport policies are devolved to Transport for London (TfL), is committed to this level of investment on an ongoing basis (Guha, 2016).

### 1.2 Barriers to investing in cycling

Barriers and opportunities may both stem from multi-level governance, which involves a variety of geographical scales and incorporates public and private organisations (Butterfield and Low, 2017). There is an interplay between broader structural processes and the actions of policy entrepreneurs (Bulkeley, 2010). Policy entrepreneurs may be needed early on to ‘kick-start’ change, but may then require the support of institutional processes and transnational networks to ensure business-as-usual (BAU) does not then take over again. Entrenched BAU may continue even where a clear case for change exists with legally binding, challenging targets. Bache et al. (2015) describe how ambitious and legally binding UK carbon reduction goals have come to constitute a symbolic meta-policy, without meaningful impact on local transport policy and practice.

The multitude of benefits conferred by an increase in cycling might lead one to ask, in Castillo-Manzano and Sánchez-Braza’s (2013) phrase: *Can anyone hate the bicycle*? Writing from Seville those authors found the answer to be yes, and the same is true in other low-cycling contexts such as England. In such contexts, cycling policies and infrastructure are contested (Lubitow and Miller, 2013), with cycling discursively marginalised and even stigmatised (Smith, 2016, Aldred, 2012). One reason may be the physical and symbolic threat such interventions pose to the car-system (Urry, 2004). Higher-cycling countries integrate cycling into their car-systems, through ‘cycle-inclusion’ in planning processes (Sagaris, 2015). However, in low-cycling contexts the normalisation of the car is associated with the de-normalisation of the bicycle, seen as illegitimate transport even (perhaps *especially*) in congested urban areas where the car offers little speed advantage.

Change in discourses and practices remain possible. In cities across Latin America, cycling infrastructure has been narrated as a claim to radically democratise urban space (Gamble et al., 2017). Across England, many practitioners support and work towards greater cycle-inclusion, networking through conferences, events and social media (Aldred et al., 2016). Their in-depth understanding of barriers and enablers are here drawn upon to construct an account of processes through which cycling investment remains de-legitimised, and processes through which this is changing, or might change. While barriers can be defined separately, Banister (2005) notes that they frequently occur together, potentially resulting in a multiplier effect influencing the outcome of an intervention. For example, in a study of 62 sustainable transport policies, Banister and Marshall (2000) found that those implementing 44 of the policies encountered three or more separate types of barrier to implementation.

This paper seeks a broad national overview of practitioner views on barriers to investing in cycling, supplemented by in-depth interviews. This is unusual within the literature on barriers to investment in sustainable transport; which tends to take a case study approach (e.g. Paget-Seekins and Muñoz, 2015, Cole et al., 2010). A national survey allows us to draw some broader conclusions about what cycling stakeholders think about the current situation for cycle planning in England. Respondents often gave additional detail in comments sections, describing experiences and perspectives, providing rich material.

## 2 Methods

### 2.1 Survey

Over four hundred people (413 from a sample of 1733—i.e. a response rate of 24%) completed an online survey on barriers to investing in cycling, open during June–July 2016. A detailed purposive sampling process was followed to ensure broad coverage of stakeholders in relevant sectors and locations, with interviewees contacted by personally addressed email. The largest single group of respondents were officers in local and regional government (e.g. for local authorities or LEPs). Others included consultants, academics, business stakeholders, and voluntary advocates and campaigners. Respondents had experience from regions across England; with for instance 13% from London governmental organisations compared to 35% from non-London governmental organisations.

The table below illustrates the diverse roles held by respondents.^2^ Aside from politicians, there were at least 20 respondents per role type:

**Table:** 

	Number of respondents	Percent (of 355)
Transport officer	146	41%
Consultant	80	23%
Other	60	17%
Academic	52	15%
Advocate	41	12%
Business stakeholder	22	6%
Politician	4	1%

We conducted a thematic analysis focused on patterns emerging from the qualitative survey data; for instance, a need to show ‘economic benefit’ from transport schemes (and a failure to do so for cycling). Themes were reviewed and organised to identify frequently cited and important issues within broader categories, making reference to Banister’s (2005) six key barriers to investing in sustainable transport. We re-frame Banister’s barriers by splitting his ‘socio-cultural’ barriers into political and stakeholder opposition. While both may be component parts of broader cultural aversion to cycling or support for the car, we found they appeared in different contexts, with different implications. Banister’s ‘legal’ and ‘land-use/topographical’ barriers were rarely cited, so we do not include these.

Our four key barriers are hence resource barriers, political barriers, institutional barriers and barriers related to stakeholder attitudes/opposition. While quotes given only relate to a minority (25) of survey participants, they have been chosen to exemplify commonly expressed themes and relationships (such as the way in which political priorities shape what are seen as funding limitations). In total 313 out of the 413 participants had provided examples of barriers, most in relation to more than one barrier.

### 2.2 In-depth interviews

While most quoted material here comes from the survey responses, we include insights from seven semi-structured stakeholder interviews conducted for the project. These are drawn upon here in providing context to the findings and discussion below: for example, detailed material about events in London.

Participants were interviewed in April and May 2016, and were chosen to provide a range of backgrounds, locations, and cycling contexts. Five were or had recently been transport planners specialising in cycling policy and planning, while one was a cycle campaigner and another a police officer specialising in vulnerable road user safety. Interviews were semi-structured around discussion of participants’ experiences whilst maintaining a logical order. Interview data helped inform the survey design; for instance, ensuring that the topics made sense to people from rural and London locations.

Five stakeholder interviewees were from England, one from Scotland, and one from Denmark; the latter two being familiar with the English context. While interviewees cannot be expected to be representative of cycling stakeholder experience in England (and survey results confirmed this) the in-depth information they provided adds insight to the discussion below. Interviews were transcribed, and as with survey data read through to explore themes, and coded to identify recurrent arguments, experiences and ideas.

## 3 Findings

### 3.1 Quantitative survey findings

From a list of eight pre-defined barriers, the top three selected were financial/funding problems, lack of political leadership, and lack of support within the transport authority in descending order. By contrast, stakeholders saw public, media and business opposition as less problematic. Other user-defined themes included:

– The bidding process as problematic (separate from the actual amounts of funding)
– Car dominance in organisations and wider culture; car (industry) lobbying
– Lack of road space to implement cycle infrastructure and (perceived) impacts of cycle infrastructure on other modes or users
– Lack of awareness of the benefits of cycling

Frequently these themes over-lapped with the pre-defined barriers; for example, many citing institutional or political barriers referred to attitudes to the car. Themes illustrated an interesting mix of structure and agency, with high-level political commitment or opposition often crucial within a broader context incorporating a range of institutional barriers.

**Table:** 

Barrier (from eight pre-defined barriers)	Percent saying barrier 'top'	Percent saying barrier in 'top three'
Financial/funding barriers	36.3%	67.3%
Lack of political leadership	29.3%	59.8%
Public opposition	7.3%	35.6%
Lack of support within transport authority	4.6%	27.1%
Transport planning tools	4.1%	19.4%
Business opposition	3.6%	16.5%
Lack of technical expertise	2.2%	15.5%
Local media opposition	1.9%	10.7%

Respondents were asked about a series of enabling factors that could help (or had helped) overcome such barriers. Overwhelmingly they choose either ring-fenced, long-term funding for cycling or high-level political support as their top enabler. Views on whether the situation was improving were mixed. Nearly half thought the situation was not changing or getting worse, while around a third thought it was getting easier to invest in cycling. People based in governmental organisations in London were over twice as likely to be optimistic as those based outside London.

### 3.2 Qualitative survey findings

We now present analysis of qualitative data on barriers to cycling investment emerging from the survey and interviews. We present these barriers categorised into four key groupings: political barriers (i.e. related to political leadership, party politics etc.), resource-related barriers (lack of funding, and problems accessing funding and finance), institutional barriers (organisational culture, support and structure, and expertise and tools available) and wider stakeholder attitudes (including the public, business, and media).

**Table:** 

Barrier	Number of examples given
Political	86 examples given of political opposition
Resources	100 examples of insufficient funding or finance
Institutional	Organisational structure or culture: 48 examples
	Tools, appraisal and modelling processes: 42
	Lack of technical expertise within organisations: 63
Wider stakeholder attitudes	Business opposition: 33 examples
	Media opposition: 33
	Public opposition: 72

#### 3.2.1 Political barriers

Among interviewees, political barriers were the most frequently cited category, raised by all. Similarly, survey respondents rated lack of high-level political support as an important barrier, although sitting just behind financial/funding barriers. The role of political barriers highlights the importance of agency and of local policy entrepreneurs willing to challenge the status quo (Bulkeley, 2010).

Many survey respondents stated that other barriers—even including funding constraints—could be overcome with a prominent local figurehead, willing to risk internal opposition or stakeholder/voter displeasure, and to leverage funding sources to support investment. However, in many places local political leaders were described as ranging from apathetic to actively hostile towards cycling investment; often more interested in motorised modes.

“Investing in cycling infrastructures or programs is something I have never heard put forward as a plan from a local mayor or other political leader for example (outside of Cambridge/London), it doesn't seem to be an important/worthy item on their agenda.”

“[A] pedestrian [and] cycling crossing 14 years in the making was struck off by the councillor with the transport brief, after 12 objections, each countered by an officer stating that this was a high volume pedestrian [and] cycling route.”

“[County council] leadership express no political will to support investment in cycling and appear not to consider cycling as a serious means of transport”

‘Not considering cycling as a serious means of transport’ highlights the often-subtle nature of political barriers to investment, and the importance of ‘car culture’ within the planning and policy field. Political barriers did not necessarily mean open hostility towards cycling investment. Instead, they could involve disinterest, inaction and/or a conflict between high-level stated commitment and a refusal to implement projects on the ground. One survey respondent noted that “our politicians will sign up to the strategy but find it very difficult to overcome local vocal opposition when the detailed schemes are proposed”. Another commented: “[Scheme] has had all its funding removed by local council yet the council is currently assisting with a strategy for cycling and getting more people active.”

Interviewees confirmed that councillors seeking re-election may be fearful of supporting plans that may become controversial. Publicly supporting and arguing for such schemes, they said, requires an expenditure of political capital that councillors may wish to reserve for issues they more strongly support. By contrast, in London in 2012 the campaign ‘Londoners on Bikes’ challenged this assumption by calling for a cycling vote (Aldred, 2014).

Survey respondents and interviewees suggested that there was little pressure on councillors and local authorities to question dominant thinking regarding mode prioritisation and the use of public space. They pointed to a continuing association of car travel with ‘economic benefit’ (see Aldred, 2012) in the minds of local decision-makers:

“Political leadership still seems to view the car as the key to economic growth. Large businesses in our town with parking problems are given sympathy and encouragement to extend their car parks.”

Organisational boundaries may aggravate political barriers. This is particularly an issue in London, where ‘Quietway’ routes^3^ often cross multiple borough boundaries, each borough controlling roads in its area. Substantial variation in the commitment of local political leaders to the routes has made successfully completing the Quietways challenging.

Overcoming political barriers was seen to require action by a driven individual or figurehead. Examples were given of such leaders, who might be technical (a zealous urban planner in a local authority) but more often political. The example of Boris Johnson, who refused to back down on cycling in the face of political opposition, was contrasted with that of a London Borough that chose not to reinstate modal filtering devices [planters restricting through motor traffic movement] after their removal by local residents. For this reason a perceived uncertainty around the commitment of new Mayor,^4^ Sadiq Khan, to cycling was raised by London-based interviewees as a potential obstacle.

Some survey respondents suggested that the very existence of a Mayoral system might be a precondition for change, enabling a policy entrepreneur to take decisive action. For example:

“Whatever you think of their politics, it's not a coincidence that Johnson/Livingstone, George Ferguson and Peter Soulsby^5^ have, as elected mayors, been able to make much more radical progress on cycling than other, more faceless LAs.”

The background to the importance of local figureheads is the broader national political context, mentioned by survey respondents and interviewees, where cycling continues to be seen as ‘local’:

“Government and DfT seem more excited with autonomous vehicles as the future of “Sustainable Transport” rather than encouraging improvements to and promotion of actual sustainable travel modes.”

Allocating road space to active modes of transport requires a strong and visible commitment from councils, some of which may pull away from robust measures through fear of local objections even if the vocal minority does not reflect the views of the wider community. Risk aversion of this kind was cited as a significant inhibitor of local action, even standing in the way of changes that could be popular with residents:

“Recommendations to implement segregated cycling facilities were overruled by elected Members, despite public support.”

The result of such risk aversion was described as frequently meaning that schemes became useless, or worse – because they created facilities so bad that they were unusable, and led to a perception that investing in cycling was pointless.

“Politicians […] feel under pressure to ease congestion on the roads and so are reluctant to support cycling schemes that reallocate road space to cyclists rather than cars. In [city], some […] routes were watered down so much from a political level that the schemes became pointless.”

Participants described how cycling investments were not safe from the political cycle, meaning that if implementation was slow, a change in political leadership could stop a scheme happening. Even if a party was re-elected, a lack of cross-party support could block implementation. A survey respondent provides an example:

“The scheme was signed off at bid stage by both main political parties. The incumbent party subsequently re-gained election but the opposition withdrew their support once the bid was successful and funding was allocated. The set-up of the political system did not seem to be conducive to allowing a strong enough leader to push it through in a timely manner.”

#### 3.2.2 Resource barriers

Financial and funding barriers loomed large in both surveys and interviews. A broader context of ongoing severe reductions in local authority spending has meant that in many authorities, many members of staff had been made redundant. Remaining officers were under huge pressure and often working outside their comfort zone. Some authorities have little transport planning function left, leading to a reliance on major consultancies which may themselves be risk averse in relation to cycling schemes.

“Limited budgets lead to limited expertise being employed to manage/design. Schemes are also compromised due to lack of funding which in turn leads to negative perception of benefit to the wider public/sponsors.”

Survey respondents criticised the allocation of major resources to road-building at a national level, and compared this with a lack of ring-fenced funding^6^ for walking and cycling:

“15bn for roads. Next to nothing for Walking and Cycling. Expecting Local Authorities to fund this when budgets being halved is unrealistic.”

Funding criteria were cited as problematic, particularly when cycling schemes compete with car-or public transport-based schemes. Historically transport cost-benefit analysis has been based on summing large numbers of small individual modelled time savings for drivers (Gössling and Choi, 2015). Cycling is typically grouped with walking as a ‘slow mode’, and often marginalised in scheme evaluation criteria. This is despite the fact that cycling journey times may be comparable to, or faster than, the car or public transport for short trips in congested urban centres.^7^ Moreover, the ‘growth’ criteria in England’s Local Growth Fund meant cycling were described as disadvantaging cycling:

“Funding process is biased towards GVA [gross value added] and this is difficult to prove for a cycling scheme.”

Organisational changes in the approach of national government towards cycling affects the way that transport schemes are assessed and funding prioritised. Outside London in particular it was suggested that the abolition of Cycling England (a national agency governing spending of resources between 2005 and 2011) and the subsequent creation of a Local Sustainable Transport Fund (LSTF), with a less explicit focus on cycling, sent a message to local authorities that cycling was being pushed down the agenda.

Centrally funded schemes were described as problematic because of the short-term nature of the funding. This was partly a problem because of the time taken to plan and build ambitious schemes, but because it threatened efforts to retain, recruit and motivate dedicated and experienced staff – contributing to institutional barriers discussed below.

“CCAG [Cycle City Ambition Grant] Tranche 2 funding only confirmed on an in-year basis. Lack of funding certainty into future financial years preventing contractual commitments being made to schemes extending over more than one financial year.”

“In [city] the issue has been less about lack of funding per se, and more about the lack of continuity in funding, which has made long term planning difficult. Cycling infrastructure schemes have had to be funded through a mix of different programmes, which have increased the administrative burden and sometimes meant that different schemes are not ‘joined up’.”

One respondent contrasted the need for steady funding to maintain an existing system, with the up-front investment needed to kick-start an entirely new system:

“Funding is allocated in pools of regular annual amounts, which works well for the maintenance and improvement of an existing system (like roads). However, when you're building a new system (like a cycle network) you need a large influx of capital up front to create a sufficient base and excitement to leverage to change behaviour (see the installation of all major motorway networks, like the US Interstate system). Then you can reduce the funding needed moving forward.”

Austerity continues to impact resources for transport: the DfT budget will be 37% lower in 2020 than it was in 2015.^8^ However, survey respondents argued that prioritisation always happens, and that the lack of political support for cycling cited above played a role. Cycling was viewed as at best an additional ‘nice to have’ and at worst, not even a legitimate mode of transport.

“At a time of very restricted public finances the priority is given to highway capacity and cycling not considered as a legitimate mode of transport hence difficult to justify additional costs.”

Finally, a tension appeared to exist between the targeted funding for cycling requested by many interviewees, and a concern expressed by some that ringfenced funding helped reinforce the view that cycling was something marginal and separate from the ‘normal’ transport system.

“[Barrier is] treating cycling infrastructure as a separate entity to general transport infrastructure which needs dedicated funding […] just like e.g. bus lanes, pavements, lighting or run-off water treatment.”

#### 3.2.3 Institutional barriers

Institutional barriers—including authority cultures and structures, and a lack of appropriate expertise, tools and models—were frequently cited. These are connected to the former, in that the short term, disconnected nature of cycling funding (due to central policy and/or local priorities) was described as hindering the capacity of local organisations to provide support for cycling.

One specific institutional issue referenced related to the growing funnelling of transport money through LEPs. Their focus on economic growth was seen in some cases as representing the continuation of a car-centric paradigm and marginalising benefits of active travel such as health gains through physical activity.

“The evidence is that LEPs think only of big economic schemes where the default is encouraging more car use (or in their minds permitting economic development through removing congestion hotspots).”

“Unfortunately our LEP is not interested in cycling (or for the most part, sustainable transport of any flavour). A recent attempt by ourselves and our Local Transport Authority to get about £2m worth of high priority cycle infrastructure schemes (linking to key employment and education destinations within our Borough) onto the LEP’s Regional Growth fund schemes pool got nowhere.”

Participants raised the issue of established approaches to traffic management obstructing the implementation of cycling schemes. Described as an ‘invisible barrier’, transport departments were criticised for dogmatically following and defending ‘guidelines laid out largely arbitrarily in the 1950s’. Respondents suggested that this placed the requirements of motorised traffic above those of other road users—for example, road works where the objective is to smooth traffic flow rather than improve the public realm. They said that it meant new opportunities were missed; for example, new housing developments built without cycling facilities.

Some respondents said that transport engineers wield a significant amount of power in local authorities because ‘they’re using numbers, they're talking about percentage capacity and all these things’.^9^ Innovative approaches in the UK context (such as cycle tracks with priority over side roads) may be viewed with suspicion. Participants commented that this reinforced the need for commitment from higher up in the political structure of organisations. The distinction between cycling and ‘real’ traffic management issues was raised: “within the highways profession cycling is seen as separate from all your other road schemes”. Decisions on road design and the allocation of space—some taken unconsciously—all affect the level of cycling provision. Cycling provision, said stakeholders, is often not considered at all until the later stages of major schemes or new developments, partly a result of the lack of formal inclusion (‘legal clout’) of cycling in planning rules, leading to substandard ‘bolt on’ cycling provisions.

Interviewees identified shortages in evidence to support cycling projects as a major stumbling point. Making the case for cycling infrastructure internally can require a strong case backed up by data to address conflict with or within transport departments. While substantial research now exists on the topic of induced demand for motoring caused by building new highway infrastructure (Næss et al., 2012) the evidence base about new cycle infrastructure and uptake remains limited. Travel demand models and traffic modelling software generally deal poorly with cycling (Twaddle et al., 2014). For this reason small case studies, with limited impact, are frequently used to estimate cycling uptake, an issue tackled by the Propensity to Cycle Tool (Lovelace et al., 2017), discussed in Section 4.

In the context of limited tools and data available at the time, interviewees cited the need to experiment by implementing schemes and learning from them as a way of demonstrating success, although this relies on sustained political and organisational will. Several participants cited the value of trial schemes using temporary materials. Whilst this can be easy, cheap and quick, temporary measures were reported as involving an element of risk and experimentation. For many local authorities, this ‘isn’t within their nature’.

Interviewees noted that national planning guidelines are often not implemented at local level, either because they are not understood or because they are poorly drafted in the first place and seen as easy to ignore. Many survey respondents, particularly in smaller local authorities, felt their transport departments did not possess the design skills necessary, and that whilst consultants (or training courses) could be bought in, these are often expensive. Frequently, planners managing cycling take on the role as one part of a greater list of responsibilities, which can leave them unfamiliar with the specific needs of cycling:

“A lack of technical expertise is limiting best practice adoption of design solutions common in other cities. Treatment of side roads along the [local A road] & around [vicinity] are examples locally.”

“[Lack of experience of cycling schemes in transport authorities] can be overcome to some extent through use of consultants but they equally have to have good experience of cycle schemes.”

Many responses claimed that in-house engineers rarely cycle and do not know what good quality cycling infrastructure looks like.

“As this is not a political priority, officers who deal with walking/cycling are not always cyclists. Often left to non-specialist project managers/transport planners to lead on design works.”

“Most cycle schemes are designed by engineers who either never cycle, or do occasionally cycle but don’t really (in my view) have a deep understanding of good design for cycling.”

One interviewee suggested that this shortage of skills should be addressed over the long term through training to ensure the next generation of engineers and planners are sufficiently equipped to challenge existing modes of thinking rather than adopt outdated approaches.

Despite the perception of transport departments as blockers of innovation, one interviewee described how in their experience this is changing, and that the last five or ten years have seen a growing flexibility in how traffic management guidelines are being interpreted and implemented (although guidelines and legislation lag behind).

#### 3.2.4 Wider stakeholder attitudes

Wider stakeholder attitudes (public, local media, business groups, etc.) were seen as problematic by survey respondents and interviewees. Here we specifically discuss these attitudes when they are cited as direct barriers to investment, as opposed to, for instance, politicians fearing such barriers. This reflects a distinction made by participants between potential schemes failing to progress (due partly, perhaps, to politicians having public opinion-related concerns) and schemes being delayed, weakened, or stopped due to the actual appearance of opposition.

Stakeholder attitudes could involve targeted opposition from specific groups (e.g. businesses, local media) or the wider public more broadly. As with cultural barriers identified within transport organisations, they were frequently described as stemming from car culture. For example:

“Anti-social parking and the widespread unwillingness to tackle it is primarily due to the widespread habits that can only be described as popular and 'public opposition' to civic sensibilities.”

Respondents cited the poor public image of cycling as contributing to public opposition to cycling schemes. This can include the view that cyclists are a special interest lobby or a group of ‘weird’ or ‘eccentric people who want to put Lycra on’.

“There is often a lack of understanding from the general public about the need for or the benefits of cycling infrastructure. Often people see it as 'the council making it harder to drive' without an understanding of why. Cycle infrastructure is also seen as something for a ‘tiny minority of Lycra warriors’.”

One survey respondent observed that the (incorrect) ‘perception is that cycling as a mode of transport gets more funding that any other’. Interviewees remarked that this view has remained largely unchanged despite the recent raising of cycling’s profile, and that the car is overwhelmingly socially and psychologically embedded, often at the expense of cycling. This was described as a vicious circle: poor quality cycling infrastructure that goes unused by cyclists but is perceived to impede traffic flow feeds anti-cycling views, pushing down cycling rates, thus further undermining support for infrastructure improvements.

The media may exploit this to set up or inflame ‘motorist vs. cyclist’ conflict. Survey respondents cited local and national news stories designed to ‘stir up anti-cycling feelings’, ‘perpetuating the “out-group” status of people on bikes’ and ‘drumming up support against schemes with incorrect information’. Examples were given of the local press being negative even where consultation responses suggested the local community was generally in favour. Whether the stance of the media is due to general editorial/proprietorial opposition to cycling schemes, concern about the impact of roadworks on traffic flow, or a desire to ‘scrutinise’ council activity, vocal opposition in media outlets may rattle decision makers and heighten the perceived riskiness of schemes.

Conversely, some survey respondents suggested that public resistance can sometimes be a more perceived than real barrier: ‘public opposition is mostly manifested through politicians’ concerns about public reactions’; ‘schemes that reduce motor vehicle capacity at junctions are rarely even attempted in anticipation of opposition’. Social and cultural attitudes among the public may be a reason for not attempting to invest in cycling, but in some cases the source of the problematic attitudes may be more accurately located within local authorities. There are certainly counter-examples of contexts where capacity for motor traffic has been removed, despite the potential for opposition (e.g. London, Leicester).

The quantitative survey results indicated that in London, wider stakeholder attitudes are more frequently cited as barriers than elsewhere. We interpret this not necessarily as meaning such barriers are greater in London, but rather the life cycle of an investment in the English context. Frequently, schemes do not make it to the design stage, and hence there is little chance for positive or negative stakeholder views to have an impact. However, where schemes do reach detailed design and implementation stages, the views of the public, media, business and other groups may become more salient. The lack of such perceived barriers outside London may hence more reflect the failure of schemes to progress than such barriers being absent.

## 4 Discussion

### 4.1 Summary of results

A complex web of interacting factors appears to be blocking the investment needed to ‘get England cycling’. Some are barriers to sustainable transport in general while some are cycling-specific. Two general barriers, specifically lack of resources and of high-level political leadership in the face of car dependency, were cited most frequently at top barriers by survey respondents. Work discussing historical failures to implement more sustainable transport policy (e.g. Doherty and Shaw, 2008) has similarly analysed the marginalisation of radical ideas throughout transport policy and planning processes, even under government with a landslide victory, a mandate for change, and a favourable economic context.

We found reference to specific barriers to investing in cycling not always faced by other sustainable modes. These interacted with general barriers, making gaining political support more challenging than for other sustainable modes. Within the UK context, and arguably North America and Australia (Daley and Rissel, 2011, Cole et al., 2010), cyclists may be perceived as a stigmatised out-group (Aldred, 2013; Christmas et al., 2010). Alongside low mode share, this creates specific problems for cycle planning. Improving pedestrian provision may face political and resource barriers, but is unlikely to be stymied by decision-makers’ views of pedestrians as weird or anti-social. Some barriers to investment are however shared with walking. Both modes have long lacked predictive planning tools and are often—as in England’s National Transport model—merely treated as a combined remainder left over after modelling motorised modes. This perpetuates a lack of predictive data not similarly experienced in planning public transport provision.

Many barriers are mutually reinforcing, such as political indifference and apathy in transport planning departments. In terms of ‘wider stakeholder attitudes’, it is not unusual in England for diverse types of transport scheme to face protest (e.g. Wall, 2002) and yet in many cases still to proceed. However, in cycling’s case, real or perceived public hostility is often aggravated by combination with other barriers. For example, improvements to cycling provision may be unthinkable if cyclists are perceived as a tiny minority of road warriors and mass cycling considered impossible. Here social and cultural barriers do not purely relate to an uninformed ‘public’ facing enlightened technocrats, but permeate institutions within a society shaped by motor dominance from the 1950s and 60s, including that technocracy.

Thinking about inter-related barriers from a ‘system dynamics’ perspective highlights the presence of feedback loops preventing change (Macmillan et al., 2016). For example, respondents gave examples of situations where cycling infrastructure was built but its quality so poor (due to political and/or budgetary barriers) that the new infrastructure was poorly used, encouraging the belief that no one wanted to cycle, and hence reducing the likelihood of building more infrastructure.

### 4.2 Overcoming barriers

The tone and outlook of survey participants and interviewees varied, and the future of cycling in the UK was described in both pessimistic and optimistic terms, with participants also framing challenges in relation to ways of overcoming them. Especially at the level of local schemes, certain individual obstacles might be reduced or removed. In one example, planners rescheduled a consultation event so that instead of taking place in a back room at a local leisure centre with only dedicated critics in attendance, it took the form of a road-show in the street, resulting in a broader cross-section of participants and an opportunity for ‘mythbusting’. Thinking in terms of processes (Macmillan et al., 2016), breakthroughs may depend on change becoming further amplified, for instance in examples given where a dedicated team succeeded in delivering a high quality intervention, leading to growth in uptake, and greater political pressure for more schemes.

Given that we found barriers were interconnected, addressing apparently more minor barriers, such as technical limitations, stakeholder and public attitudes, might help challenge entrenched political obstacles. For example, new tools such as the Department for Transport’s Propensity to Cycle Tool (PCT^10^) or Transport for London’s Analysis of Cycling Potential can help local policy-makers by providing detailed evidence for local investment strategies (Lovelace et al., 2017). Such tools are important because they map cycling *potential* rather than existing cycling; crucial where cycling levels are currently low and skewed. Developing a clear vision for a local cycle network—both specific routes and quality thresholds—can prepare authorities to take advantage of new funding sources, and help secure buy-into minimise stakeholder opposition that may otherwise appear later. Dissemination of successful local examples can help build the case for larger-scale change.

Many interviewees suggested trialling—testing an intervention relatively cheaply before deciding to expand and/or make permanent if successful—to overcome inertia in the planning system. Education of officers and politicians may help overcome misunderstanding and fear. Policy that successfully challenges car culture and demonstrates what can be achieved may lay the groundwork for further sustainable transport investment by reducing the perceived political risk. For example, there has been a recent growth in ‘close pass’ policing^11^ by local police services in England, the impacts of which have yet to be researched. Finally, there are lessons for advocacy in the data even if points made do not specifically address advocates. For instance, the need to narrate cycling in the context of other policy goals and to ensure that policy-makers do not focus only on existing cyclists, but rather on the benefits offered by a shift towards mass cycling.

### 4.3 Processes of reinforcement and change

The findings should be seen in the context of the ongoing dominance of a narrative privileging ‘the economic’; often conflated with the car (Aldred, 2012), and an ongoing ‘austerity’ stripping authorities of resources and expertise. However, organisations at different levels retain some level of freedom to redefine ‘the economic’ and even to privilege goals seen as not ‘economic’. This is influenced by stakeholders; for instance in London TfL was affected by the growing publicity given to cyclist deaths, which generated severe reputational risks (Macmillan et al., 2016), as might growing awareness of the impact of air pollution on health. Stakeholders in TfL and other organisations have also sought to redefine cycling as economically productive (see e.g. Grous, 2011) although discourses, representations, tools, and techniques to do this remain under-developed.

The data suggests that English authorities still struggle to achieve ‘cycle-inclusion’ (Sagaris, 2015) as found in higher-cycling contexts. Even within London, which has done much to advance tools and models for cycle planning, progress remains patchy across boroughs and schemes, rather than cycling being treated as integral to planning processes. Hence, even where (i) some high-level political support exists, (ii) organisations are relatively well-resourced, and (iii) cycling has an organisational base including the development of specialist planning tools, cycling’s legitimacy remains precarious. In London, specific schemes have become subject to vociferous anti- campaigns (see e.g. the ‘mini-Holland’ interventions in Enfield, Kingston, and Waltham Forest, which have sought to improve cycling and walking conditions in Outer London boroughs: Rogers, 2016). This can in turn threaten political support for such schemes, particularly given cycling has traditionally been marginalised and de-normalised as a mode of transport.

### 4.4 Study strengths, weaknesses, and future research

A strength of this study is that there are few similar pieces of research seeking out nation-wide stakeholder views on cycling investment. More such studies in other contexts, and indeed cross-national comparative studies, would also be valuable. One weakness might be the purposive as opposed to random sampling, although it is hard to see how a diverse and disparate stakeholder community might be randomly sampled. Stakeholders surveyed here are largely (not exclusively) supportive of cycling investment, and provide a partial and sometimes polemical analysis. Where they are from local authorities, they tend to be officers rather than politicians, as noted earlier. However, they represent people who have actively tried to build cycling infrastructure in the English context, people who often care greatly about cycling and have provided not only quantitative data but also often in-depth descriptions of their experiences. Many have significant insights into the barriers discussed here, which this paper tries to synthesise.

A complementary piece of future research might survey sustainable transport practitioners more broadly on barriers to investing in different sustainable modes, thereby allowing drawing of comparisons between barriers and ways of overcoming them. This might allow a more in-depth discussion of lessons to be learnt from providing from different modes, and how planning and improving provision for all non-car modes might better be integrated at network level. Another useful piece of further research might focus on local politicians, whose response rate was low, by contrast to local authority officers, consultants, and academics. Other methods to elicit their perspectives should be tried; for example, analysis of ‘videocasts’ and/or transcripts of local council meetings where cycling or sustainable transport are discussed. Such methods could also provide different insights to those gleaned from an online survey. Finally, researchers might seek to include variables related to these barriers and enablers within models predicting infrastructure development and/or cycling uptake (see e.g. Hackl et al., 2017).

### 4.5 Conclusion

The results of this research may seem rather negative to those advocating investment in cycling. However, it is only by identifying barriers accurately that they can be overcome. Despite the entrenched nature of many barriers, international evidence does suggest that they can be successfully tackled. Many cities are seeing a reduction in motor traffic and an increase in cycling rates (Buehler et al., 2017, Lanzendorf and Busch-Geertsema, 2014, Kim, 2015). Lessons may be learnt from the successful promotion of other sustainable modes. For example, London since the 1990s has seen an impressive mode shift away from the car, towards public transport and particularly the bus, despite its image as a low status mode (Ellaway et al., 2003).

Within England, positive examples exist where cities have invested in cycling, with cities including London and Leicester cited as having managed to some extent to overcome funding and political barriers, despite ongoing challenges. In London, for example, powerful opponents of new cycling routes included Canary Wharf Group and the Licensed Taxi Drivers’ Association. However, without a clear national lead, evidence presented here suggests that it will be challenging to translate London’s experience into smaller urban and rural contexts where car culture is more entrenched and funding more limited.

The analysis of stakeholder perceptions highlighted opportunities for progress outside London, however, which may be relevant to contexts outside England experiencing similar challenges. The research presented in this paper shows that many of the local barriers to effective investment in cycling are systemic. This insight suggests that both sufficient long-term funding and bold political leadership are needed to shift decisively away from the current ‘vicious circle’ of cycling policy (no money > poor facilities > no cycling > cycling marginalised > perceived political risk > no money).

If barriers to cycling are tackled effectively, based on the best available evidence, this could lead to an equivalent ‘virtuous circle’, in which money spent well (using new tools such as the PCT) leads to better facilities that get used, leading to political support and more money. Regardless of which element in this positive feedback loop initiates systemic change towards strategic planning for cycling, one thing is clear from practitioners: high quality infrastructure constructed where it is needed, in combination with cycling-friendly policies, can help build a strong voice, locally and nationally, demanding more cycling investment.

## Footnotes

1 https://www.gov.uk/government/collections/local-growth-deals.

2 Respondents could pick more than one; 355 answered this question.

3 The brand given for cycle routes primarily built away from main roads.

4 At the time that interviews and the survey were conducted.

5 Previous mayors of London and Bristol, and current – at time of writing – mayor of Leicester.

6 I.e. funding that can only be used for its specified purpose.

7 See e.g. for London https://tfl.gov.uk/info-for/urban-planning-and-construction/planning-with-webcat/webcat?scenario=2031%20(Forecast)&Type=TIM.

8 https://www.theguardian.com/politics/2015/nov/25/spending-review-transport-deepest-budget-cut.

9 See below for the role of data and evidence.

10 See www.pct.bike.

11 Where plain-clothed police officers on bikes are used to stop drivers passing them too close, and educate offenders.
